# Effect of heavy cigarette and water pipe smoking on antioxidants and lipids in Sudanese male smokers: a case-control study

**DOI:** 10.4314/ahs.v22i3.15

**Published:** 2022-09

**Authors:** Ahmed M Ahmed, Amna M Ibrahim

**Affiliations:** 1 Department of Clinical Laboratory Sciences, Faculty of Applied Medical Sciences, Taibah University, AL Madinah, Saudi Arabia; 2 Faculty of Medicine, Omdurman Islamic University, Khartoum, Sudan

**Keywords:** Smoking, antioxidants, cigarette smoking, CS, water pipe smoking, WPS

## Abstract

**Background:**

Tobacco smoking is a source of many toxins such as free radicals, mutagenic substances as well as cause for developing cardiovascular diseases (CVD), particularly atherosclerosis. This study aims to assess the impact of smoking on antioxidants in Sudanese male smokers.

**Methods:**

Cases were 85 and 48 men who smoke cigarettes (CS) and water pipe (WPS) respectively and they were compared with matching 50 non-smoking controls. Blood samples were collected and following parameters: Glutathione peroxidase, Superoxide dismutase, Total cholesterol, Triglyceride, LDL, HDL, Paraoxinase, and Malondialdehyde were measured.

**Results:**

There were no significant differences in biochemical parameters between light CS and WPS compared to controls. In heavy smokers of both WPS and CS, the TC, TG, LDL, and MDA were higher than controls (p>0.05), GPx, SOD, HDL, and PON were lower in smokers than controls (p>0.05). In both groups of smokers; HDL, GPx, SOD, and PON were inversely correlated with duration of smoking (p>0.05), also, HDL was positively correlated with SOD and GPx (p>0.05). Moreover, GPx and SOD were correlated with each other in both groups of smokers (p>0.05).

**Conclusion:**

In Sudanese male smokers' biochemical profile disturbances suggest that heavy smoking was leading to developing CVD, particularly WPS.

## Introduction

Tobacco smoking is a significant reason for death and disability internationally. WHO counts 5 million deaths annually because of tobacco use^1^. Death caused by smoking regarding CVD might have been preceded by subclinical cardiovascular abnormalities, for example, injury and raised biomarkers in asymptomatic subjects^2^ ^3^,^4^.

Tobacco use poses the greatest hazards and challenges worrying public health in Africa, because of the expansion in utilizing Cigarette smoking (CS) and Water Pipe Smoking (WPS), both of which have a significant influence on public health and are considered causative factors of chronic diseases such as cancer and coronary artery disease 5.

Since cigarette smoking is expanding in sub-Saharan Africa, especially in men, which increments many dangers compromising public health^6^.

In Sudan, about 20% of people use various types of tobacco, with about 8% of them smoking cigarettes. Tobacco use was found to be 2% among children and young adults, and cigarette smoking was found to be 12% among adults aged 18 and up. The majority of cases were found in urban areas rather than rural areas^7^. Moreover, the utilization of tobacco in the form of CS, WPS, and Tombak (snuff) is largely spread among Sudanese young adults and adolescents^8^–^10^.

CS is associated with the progression of the pathogenesis of numerous illnesses, including atherosclerosis and cancer, because it produces a significant amount of free radicals and reactive oxygen species (ROS)^11^, These free radicals and ROS can damage tissues through oxidative pressure due to an imbalance between the reduced amount of antioxidant and raised free radicals^12^.

WPS is a popular form of smoking in Sudan now and inquisitively among people who smoke because of its varied flavors types, and misperception that it is less risky to diverge from cigarette smoking considering the way that the usage of water as a filter^13^. It was named locally as Shisha and in a recent cross-sectional study done on school students; the rate of smokers was equivalent to 13.4% over both sexes^14^. New research details that WPS could cause oxidative, inflammatory, and mutagenic effects on human health that might prompt chronic sicknesses. Moreover, WPS contains a high proportion of nicotine and tar that causes serious and consistent CVD^15^.

The current investigation was undertaken to determine the association between CS and WPS on the levels of lipids, Glutathione peroxidase (GPx), Superoxide dismutase (SOD), Malondialdehyde (MDA), and Paraoxinase enzyme (PON), which compared with non-smoking subjects.

## Methods

### Participants

In this case-control study, eighty-five cigarette smokers' (CS) men (age 32±0.25 years), with various categories of smoking; sixty-five were light smokers and twenty were heavy smokers (for the mean period of 12.6±0.46 years). Light and heavy smokers were classified as follows: light smoking has been described as smoking less than 10 cigarettes/day^16^, while heavy smoking might have been described as smoking ≥25 cigarettes/day^17^. Compared to forty-eight water pipe smoker (WPS) men (age 33±0.98 years), with different categories of smoking; thirty-six were light smokers and twelve were heavy smokers. Light smoking was smoking about one time/week, and heavy smoking was smoking 1–2 times/day^18^. Both groups (CS and WPS) were compared with fifty nonsmoking healthy men as a control group with age ranged between 20–50 years with a mean age of 33±0.6 years. Controls were close associates of the smokers who sit with them during the smoking period, therefore sometimes they may be passive smokers. They were of comparable age and gender to the cases. The clearance for this study was taken from the Institutional Review Board (IRB) of the faculty of applied medical sciences at Taibah University, Madinah, Saudi Arabia, which follows the measures of the declaration of Helsinki and all of its amendments. All participants were informed of the aim of this study and then they concurred as volunteers and signed consent. Exclusion criteria included diabetes mellitus, thyroid diseases, patients with chronic renal/liver disease, cancerous diseases, anemia, and those who use antioxidant/vitamin or mineral supplements.

### Biochemical parameters

Fasting blood samples were taken from study groups in plain tubes and the serum was separated close to collection time. Total Cholesterol (TC), Triglyceride (TG), Low density lipoprotein (LDL), and High density lipoprotein (HDL) were measured with a full auto-analyzer (Hitachi 704, Roche Diagnostics Switzerland). Serum antioxidant, GPx level was measured according to changes in nicotinamide adenine dinucleotide phosphate (NADPH) which was read at 340 nm spectrophotometrically, Paglia et al^19^, and SOD was determined by using the principle of nitro blue tetrazolium (NBT) reduction rate (Durak et al^20^. The Paraoxonase (PON) enzyme level was measured by Elabscience's ELISA kit (Sandwich-ELISA principle); and we follow full ELISA protocol which was previously described by Ahmed^21^. Malondhyde (MDA) was measured using thiobarturic acid reactive substances (TBARS) according to Ahmed et al^22^.

### Statistical analysis

The data was analyzed with SPSS version 21 (IBM Corporation, Armonk, NY, USA). The normality of our data was tested using the Shapiro-Wilk Test, and the result was greater than 0.05, indicating that our data was normally distributed. For comparisons of three groups, ANOVA followed by Tukey's post-hoc test was conducted. An unpaired t-test was used to determine the difference between groups and Pearson correlation were used to determine the correlation between two sets of data. For comparisons of frequencies data, the chi-square/Fisher's exact test was applied. A P<0.05 indicated significant differences.

## Results

**Figure 1:**
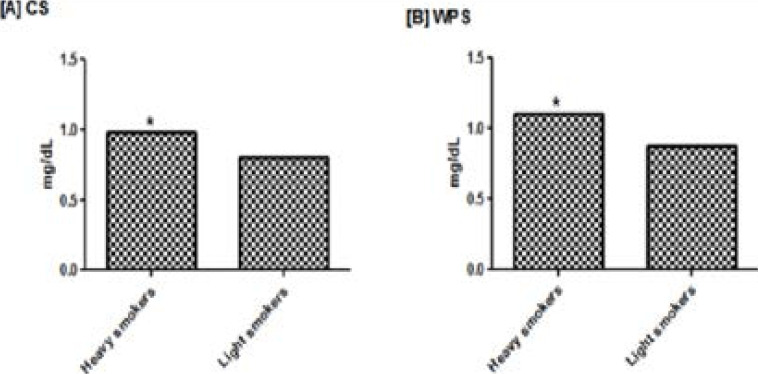
Basal serum MDA concentration in heavy and light smokers in both CS and WPS. * Significant at p>0.01. CS: Cigarette smokers. WPS: Water pipe smokers. MDA: Malondialdehyde.

A total of one hundred and thirty-three male smokers were included in this study, eighty-five subjects were CS and forty-eight were WPS. Table 1 shows the socio-demographic characteristics of smokers, isolated and altogether. In which there was no significant difference. Table 2 shows the demographic and biochemical profile of smokers contrasted with controls. The duration of smoking in CS was higher than that of WPS (p < 0.001). In addition, the mean level of antioxidants GPx and SOD were lower in both groups of smokers compared to controls (p < 0.001), and lower in WPS compared to CS (p < 0.001). Moreover, PON was lower in both groups of smokers than controls (p < 0.001), and lower in WPS than in CS (p < 0.01). Table 3 shows the comparison of the mean levels of lipid profiles and antioxidants between heavy and light smokers in both CS and WPS, in which TC, TG, and LDL were higher in heavy smokers than in light smokers (p < 0.05). In addition, HDL was lower in heavy smokers compared to light smokers in CS (p < 0.05). Moreover, with respect to antioxidants mean levels, GPx and SOD were lower in heavy smokers compared to light smokers, (p < 0.05). Table 4 shows the correlation between age, duration of smoking and antioxidant levels. HDL, GPx, SOD, and PON were contrarily correlated with the duration of smoking in both CS and WPS (p < 0.05). In addition, HDL was positively correlated with SOD and GPx in both groups of smokers (p < 0.05). Moreover, GPx and SOD were positively correlated with each other in both groups of smokers (p < 0.05). Figure 1 shows the comparison of MDA level in heavy and light smokers; MDA was higher in heavy than light smokers (p < 0.01).

**Table 1:** Socio-demographic characteristics of smokers groups

	Cigarette smokers n = 85	Water pipe smokers n = 48	Total 133	P value
Age groups				
< 20 years	6	3	9	
21–30 years	33	15	48	
31–40 years	26	22	48	0.47
41–50 years	17	6	23	
> 50 years	3	2	5	

Residence				
Urban	62	28	90	0.08
Rural	23	20	43	

Education level				
Illiterate	6	8	14	
School	36	17	53	0.14
University	30	20	50	
Postgraduate study	13	3	16	

Marital status				
Single	38	17	55	
Married	12	10	22	0.60
Widow	9	4	13	
Divorce	26	17	43	

Occupation				
Employee	32	18	50	
Worker	19	14	33	
Tradesman	15	10	26	0.68
Farmer	3	1	3	
Student	16	5	21	

Total	85	48	133	

P value was determined with chi square test, for low number (<5) fisher exact test was applied.

**Table 2:** demographic and biochemical profiles of smokers both groups compared to controls

Character	Cigarette smokers n = 85	Water pipe smokers n = 48	Control subjects n = 50	P value
Age (years)	32±2.3	33±6.7	33±4.2	0.31
Duration (years)	12.6±4.2	8.7±3.4	-	<0.001
Biochemical data				
TC (mmol/L)	3.8±1.1	3.8±0.9	3.5±0.6	0.17
TG (mmol/L)	1.1±0.5	1.2±0.7	1.0±0.5	0.27
LDL (mmol/L)	1.6±0.7	1.8±0.9	1.5±0.5	0.15
HDL (mmol/L)	1.45±0.3	1.43±0.1	1.5±0.1	0.14
Antioxidant profiles				
GPx (U/L)	112.8±23.1*	102±15.9*^a^	132.3±31.8	<0.001
SOD (U/L)	8.4±1.7*	6.8±2.1*^a^	11±1.9	<0.001
PON enzyme (ng/mL)	60.8±7.7*	56.1±8.4*^a^	97.9±8.2	<0.001
MDA (mg/dL)	0.89±0.4	0.91±0.2	0.78±0.2	0.07

Abbr eviations: n: number. TC: total cholesterol. TG: triglyceride. LDL: Low density lipoprotein cholesterol. HDL: High density lipoprotein cholesterol. GPx: Glutathione peroxidase. SOD: Superoxide dismutase. PON: Paraoxinase. MDA: Malondialdehyde.

Data represent as mean ± standard deviation (SD).

* Significantly different to controls (P>0.01)

a Significantly different to cigarette smokers (P>0.01).

**Table 3:** Lipids and antioxidants levels in light and heavy both groups of smokers

Character	Cigarette smokers n = 85		P value	Water pipe smokers n = 48		P value
	
	Light smokers (<10/day) n=65	Heavy smokers (≥25/day) n=20		Light smokers (1–2 times/week) n=36	Heavy smokers (1–2 times/day) n=12	
TC (mmol/L)	3.6±0.4	4.1±0.58	0.02	3.7±0.78	4.4±1.2	0.02
TG (mmol/L)	1.0±0.16	1.6±0.26	<0.001	1.1±0.18	1.7±0.2	<0.001
LDL (mmol/L)	1.6±0.16	1.9±0.26	<0.001	1.7±0.24	2.0±0.62	0.02
HDL (mmol/L)	1.48±0.08	1.2±0.09	<0.001	1.45±0.18	1.1±0.62	0.003
GPx (U/L)	123.5±32.2	104.7±16.5	0.01	115±23.4	98±18	0.03
SOD (U/L)	8.3±1.6	7.1±0.9	0.008	7.2±0.6	6.1±0.7	0.007
PON (ng/mL)	64.2±9.2	57.7±10.2	0.008	59.0±7.2	52.5±8.6	0.009

Data represent as mean ± SD. P value was calculated by unpaired t-test. p.0.05 consider significant.

Abbreviations: n: number. TC: total cholesterol. TG: triglyceride. LDL: Low density lipoprotein cholesterol. HDL: High density lipoprotein cholesterol. GPx: Glutathione peroxidase. SOD: Superoxide dismutase. PON: Paraoxinase.

**Table 4:** Pearson correlation coefficient between lipids, duration of smoking and antioxidants in both smokers' types

Character	Cigarette smokers				Water pipe smokers			

	Age	Duration of smoking (r2)	GPx	SOD	Age	Duration of smoking (r2)	GPx	SOD
TC	0.09	0.12	0.14	0.11	0.11	0.12	0.22	0.22
TG	0.03	0.08	0.13	0.10	0.07	0.09	0.15	0.11
LDL	0.07	0.11	0.22	0.24	0.08	0.09	0.20	0.17
HDL	0.26	-0.33*	0.36*	0.37*	0.34*	-0.42*	0.46*	0.38*
GPx	0.41*	-0.76*	-	0.81*	0.48	-0.85*	-	0.88*
SOD	0.34*	-0.78*	0.81*	-	0.4	-0.87*	0.88*	-
PON	0.12	-0.53*	0.16	0.21	0.12	-0.57*	0.21	0.20
MDA	0.18	0.21	0.16	0.19	0.09	0.23	0.19	0.20

* P< 0.05.

Abbreviations: n: number. TC: total cholesterol. TG: triglyceride. LDL: Low density lipoprotein cholesterol. HDL: High density lipoprotein cholesterol. GPx: Glutathione peroxidase. SOD: Superoxide dismutase. PON: Paraoxinase. MDA: Malondialdehyde.

## Discussion

In the current study, the modulation of lipid profiles (TC, TG, LDL, and HDL), two antioxidants (GPx, and SOD), oxidative biomarker (MDA), and PON enzyme among CS and WPS were examined. The most striking results were higher levels of TC, TG, LDL, and MDA concomitant with low levels of HDL, GPx, SOD, and PON in heavy smokers of both CS and WPS than in light smokers. In the current finding, low antioxidants and PON enzyme reflect the high amounts of free radicals which may generate oxidative stress. Cigarette smoking is a leading factor for oxidative stress and cytolysis^23^. This finding is supported by other studies around the world^11^,^24^–^26^. The molecular mechanism of association between cigarette smoking and CVD is not known yet, but it could be due to the induction of oxidative stress in the cardiovascular system, leading to many bad effects 27,28.

In both cigarettes and WPS, many toxins could be delivered harmful to human health, such as carbon monoxide, nicotine, polyaromatic hydrocarbons, volatile aldehydes, and tobacco-specific nitrosamines^29^, one session smoke of WPS (30–60 min) the subject was inhaled over ≤40-liter smoke compared to ≤1-liter for cigarette smoker^15^. In our study, we found that heavy WPS were more affected compared to CS based on the evidence of biochemical outcomes such as antioxidants, lipids, PON, and MDA, which are very high in WPS groups. This proposed WPS is a serious form of smoking because of the quantity of bad materials, long period of smoking sitting, and more amount of smoke breathed; this is agreed with the finding of Pratiti and Mukherjee^15^ who showed WPS induces oxidative stress by impairing the function of endothelial vasodilator and its repairing mechanisms, that elevated transcriptional expression of matrix metalloproteinase an immune response regulator thereby inducing inflammatory and inactivation of cellular growth. Oxidative stress plays a key role in the progression of chronic diseases such as CVD, particularly atherosclerosis, which is developed by an imbalance between smoking-induced free radicals (reactive oxygen species) and antioxidant defense mechanisms, and our outcome documented this finding and suggested the serious risk of WPS more than non-smokers and decreased of antioxidants levels which correlated with the period of smoking, and Yalcin et al, supported that^30^. In agreement with the previous review concerning the association between WPS and coronary artery disease (CAD), the author found positive evidence of developing CAD and other CVD in smokers. Our study supports this finding because of positive evidence of CVD including high lipids, high oxidative biomarker (MDA), low antioxidants, PON and HDL suggested the risk of our both smokers groups CS and WPS^31^. In agreement with the previous prospective cohort study done in Sudanese CS, the authors concluded an association between CS and myocardial infarction and a correlation between the risk of myocardial infarction and smoking duration^32^. Among the limitations of the current research include, besides low sample size, comparison of period and amount of smoking for both CS and WPS to estimate the real differences between exposure to CS and WPS in biochemical indicators, and estimate the toxins materials in both smoker's groups such as carbon monoxide, nicotine, polyaromatic hydrocarbons, volatile aldehydes, and tobacco-specific nitrosamines. So we recommend another study with a large sample size.

## Conclusion

Sudanese heavy smokers' males had higher levels of lipids, “including bad lipoprotein (LDL)” and an oxidation marker (MDA). However, they had lower levels of good lipid (HDL), PON enzyme, and antioxidants (SOD and GPx), indicating that they were at risk of developing CVD, particularly in WPS. The progression of the smoking period was directly correlated with these disturbances, and WPS were at higher risk compared to CS.
